# Serum levels of M-CSF, RANKL and OPG in rats fed with Kashin-Beck disease-affected diet

**DOI:** 10.1186/s13018-014-0078-3

**Published:** 2014-08-20

**Authors:** Denglu Yan, Fuxing Pei, Yancheng Song

**Affiliations:** 1 Nanshan Hospital of Shenzhen University, Shenzhen 518052, People’s Republic of China; 2 West China Hospital, Sichuan University, Chengdu 610065, People’s Republic of China; 3 Third Hospital of Nanfang Medical University, Guangzhou 510630, People’s Republic of China

**Keywords:** Kashin-Beck disease, Macrophage colony-stimulating factor, Osteoprotegerin ligand receptor activator of NF-kappaB ligand, Osteoprotegerin

## Abstract

**Objective:**

There were no studies on the macrophage colony-stimulating factor (M-CSF), receptor activator of NF-kappaB ligand (RANKL) and osteoprotegerin (OPG) in the pathogenesis of Kashin-Beck disease (KBD). The objective of the present study was to investigate the serum M-CSF, RANKL and OPG in rats fed with KBD-affected diet.

**Methods:**

Ninety Wistar rats were divided into five groups. The rats received standard commercial feed with or without T-2 toxin additive, low protein feed with or without or T-2 toxin additive and the KBD-affected feed. The serum bioactivity of M-CSF, RANKL and OPG was tested by enzyme-linked immunosorbent assay.

**Results:**

The serum levels of M-CSF in E group rats were higher than those in the other groups in the five groups (*P* < 0.01). The serum levels of RANKL and OPG in E group rats were highest in the five groups and have significant difference compared to the other groups (*P* < 0.05).

**Conclusions:**

The molecule of M-CSF, RANKL and OPG may be involved in the regulation of epiphyseal plate injury and repair in KBD, and its participation in the pathogenesis of KBD should be studied in the future.

## Introduction

The important function of bone can be easily recognized in day-to-day life, where millions of people suffer from bone disease such as osteoporosis, which is, in part, caused by an imbalance between bone formation and bone resorption [1],[2]. The better understanding of the regulation of bone formation will provide new insights into the molecular mechanisms of Kashin-Beck disease (KBD), which usually afflicts children between the ages of 5 and 13 years with epiphyseal plates showing focal or irregular premature closure, wherein in the normal bone formation, endochondral ossification ends up about 20 years after birth in humans [3]-[5]. Its initial pathologic changes are multiple degenerative and necrotic lesions within the growth plate cartilage. The secondary pathologic findings, visible on radiographs, are repairing and remodeling around the necrotic foci of the cartilage of the metaphysis, bone end and epiphysis [6]-[9]. With the development of modern molecular biology technology, there has been a focus on serum biomarkers as tools in evaluating cartilage destruction [10]. However, there is little understanding of the expression of these biological molecules and the relationship with possible pathogenic factors of KBD.

Based on epidemiological investigations, three main hypotheses for the etiology of KBD are considered to be selenium deficiency in food, hypoalimenation in food and severe contamination of food by fungal mycotoxins [11]-[15]. The high altitude of the A'ba Autonomous Region of Sichuan province, just as Tibet, and the extremely severe weather conditions affect the availability of natural resources and the use of land. Due to high moisture content, the grains were particularly susceptible to mould and infected by fungi [16]-[18]. Cereal grain contamination by *Fusarium* spp. and *Alternaria* sp. in endemic areas of China, and cereal samples in high incidence areas of KBD were reported to be more heavily contaminated with trichotecenes (T-2 toxins) when compared to those in low incidence areas [19],[20]. Moreover, T-2 toxin can bind to the eukaryotic 60 S ribosomal subunit causing inhibition of protein, DNA and RNA synthesis. Although T-2 toxin can inhibit the proliferation of cartilage cells and reduce synthesis of proteoglycan and collagen in epiphyseal plate through metaphyseal rich and open capillary network, the fact is that the blood system was the first target of T-2 toxin involvement and bone marrow may be the main network place other than the epiphyseal plate. However, there has been little knowledge about the blood system and bone marrow biological process that have been regulated by T-2 toxin and the relationship with possible pathogenic factors of KBD.

It is generally believed that bone marrow contains progenitor cells both of osteoblasts and osteoclasts [21],[22]. On the basis of bone marrow biological process regulated by T-2 toxin, the microenvironment for progenitor cells in bone marrow may play an important role in the pathological changes of KBD [23]. At the molecular level, several factors are known to cooperate in regulating the sequential steps of endochondral bone formation in bone marrow, including the macrophage colony-stimulating factor (M-CSF), receptor activator of NF-kappaB ligand (RANKL) and osteoprotegerin (OPG) [24]-[26]. RANKL is a transmembrane ligand expressed in osteoblasts and bone marrow stromal cells and produced by T cells. Following binding to RANK, a receptor vital for osteoclast differentiation, activation and survival, RANKL induces osteoclastogenesis in a pathway which synergizes with signals derived from M-CSF. OPG, also produced by osteoblasts and marrow stromal cells, lacks a transmembrane domain and acts as a decoy receptor for RANKL, thus regulating bone metabolism [27]. In view of the bone formation and KBD which usually afflicts children between the ages of 5 and 13 years with epiphyseal plates with focal or irregular premature closure, M-CSF/RANKL/OPG roles may be involved in the regulation of epiphyseal plate injury and repair in KBD. However, no paper has been published in this respect. In this study, the serum concentrations of M-CSF, RANKL and OPG were tested in order to evaluate their participation in the pathogenesis of KBD.

## Materials and methods

Ninety weanling Wister rats (half were males and females, 4 weeks of age and with weight of 60 to 70 g) were obtained from the Center of Laboratory Animals of West China hospital, Sichuan University, Chengdu, Sichuan, People's Republic of China. The rats' rooms were maintained at a temperature of 22°C with relative humidity of 40%–70% and 12-h light/dark cycle. Prior to initiation of dosing, all rats were quarantined for 1 week and evaluated for weight gain and any gross sign of disease or injury. After quarantine, the rats were randomly divided by a computerized blocking procedure into five groups (every group has 18 rats). Group A was fed with a normal diet as control, group B was fed with a normal diet and T-2 toxin, group C was fed with a low nutrition diet and T-2 toxin, group D was fed with a low nutrition diet and group E was fed with a KBD-affected diet. All rats were supplied with tap water that is freely accessible at all times. Use of animals in this study was in accordance with NIH publication 85-23 ‘Guide for Care and Use of Laboratory Animals’ (NRC, 1996). The experiment was approved by the Animal Ethics Committee, West China school of Medicine, Sichuan University.

KBD-affected diet was made on the basis of the KBD-affected family dietary pattern by the wheat and corn samples, which were collected in October 2008 from KBD-affected family which was located in Jinchuan county of the A'ba region, Sichuan, People's Republic of China. Normal diet was based from the commercial rats' diet. The details of the dietary pattern and nutritional constitution of diet can be seen in our previous report [28],[29]. Every rat has been feed sufficiently to avoid conflict caused by hunger (20 to 30 g diet for each rat everyday). T-2 toxin was purchased from Trilogy Analytical Laboratory (Washington, MO, USA) at a purity of 99%; crystalline T-2 toxin was dissolved in absolute ethanol and diluted with 0.9% normal saline. The T-2 toxin was continuously administered intragastrically over 5 days in 1 week at levels of 0.04 mg/kg/day.

At 4, 8, and 12 weeks, six rats from each group were randomly sacrificed after collecting blood samples. After collection, blood samples were centrifuged and the serum obtained was stored at −80°C up to measurement. Serological investigations were carried out at the end of the study in all serum samples taken at the different time points. Assay were carried out to determine serum levels of M-CSF, RANKL and OPG by a sandwich enzyme-linked immunosorbent assay (ELISA), utilizing two monoclonal antibodies directed against separate antigenic determinants on the rat M-CSF, RANKL and OPG. The kits for the assay were provided by Adlitteram Diagnostic Laboratories Inc. (San Diego, CA, USA).

### Statistical analyses

Statistical analyses were performed using SPSS Version 16.0 software. The data were expressed as the mean ± SD and were analyzed using analysis of variance (ANOVA). In the case of heterogeneity of variance, transformations were used to stabilize the variance. Results were considered statistically significant if the *p* value was less than 0.05 for continuous variables.

## Results

ELISA analyses were used to determine the serum levels of M-CSF, RANKL and OPG in experiment rats (Table 1). The serum levels of M-CSF were similar in the control group and low nutrition group rats in the experience course at three time points (*P* > 0.05). The serum levels of M-CSF in E group rats were highest in the five groups at 4 and 8 weeks (411.85 ± 123.73 pg/ml versus 589.62 ± 147.85 pg/ml, respectively) and have significant difference compared to the other groups (*P* < 0.01). However, there were no differences in every group at 12 weeks (*P* > 0.05). C group rats just as the E group have the highest serum levels of M-CSF at 8 weeks and have significant difference compared to those at 4 weeks (376.30 ± 128.19 pg/ml versus 151.27 ± 81.22 pg/ml, *P* < 0.01).

**Table 1:** Serum levels of M-CSF, RANKL and OPG

**Item**	**Time (weeks)**	**A**	**B**	**C**	**D**	**E**
MCSF (pg/ml)	4	234.08 ± 82.08	116.61 ± 92.67	151.27 ± 81.22	246.76 ± 97.41	411.85 ± 123.73
	8	231.36 ± 85.53*	138.82 ± 95.76*	376.30 ± 128.19*	259.64 ± 88.97*	589.62 ± 147.85**
	12	232.56 ± 88.13	228.27 ± 76.63	250.77 ± 95.40	248.09 ± 93.52	258.87 ± 89.86
RANKL (ng/ml)	4	0.034 ± 0.025#	0.079 ± 0.038#	0.138 ± 0.059#	0.068 ± 0.017#	0.228 ± 0.085##
	8	0.041 ± 0.017	0.084 ± 0.041	0.178 ± 0.022	0.052 ± 0.028	0.160 ± 0.059
	12	0.058 ± 0.013	0.072 ± 0.038	0.153 ± 0.027	0.053 ± 0.011	0.105 ± 0.042
OPG (ng/ml)	4	1.415 ± 0.312※	1.492 ± 0.391※	1.734 ± 0.778※	1.324 ± 0.773※	2.688 ± 0.615※※
	8	1.665 ± 0.597	1.542 ± 0.363	1.376 ± 0.369	1.486 ± 0.598	1.359 ± 0.517
	12	1.672 ± 0.476	1.403 ± 0.624	1.241 ± 0.134	1.461 ± 0.708	1.288 ± 0.612

There were difference in the groups of * compared to **, # compared to ## and ※ compared to ※※, *P* < 0.01.

Mean ± SD.

The serum levels of RANKL in E group rats were highest at 4 weeks (0.228 ± 0.085 ng/ml), which have significant difference compared to the other groups at 4 weeks and compared to 8 or 12 week levels in E group (*P* < 0.01). At 8 and 12 weeks, C group rats have the highest serum levels of RANKL in five groups (0.178 ± 0.022 ng/ml versus 0.153 ± 0.027 ng/ml), which have significant difference compared to A, B and D (*P* < 0.05) and no significant difference compared to E (*P* > 0.05).

The serum levels of OPG were 2.688 ± 0.615 ng/ml in E group at 4 weeks, which was highest in the five groups and have significant difference compared to the other groups (*P* < 0.05). However, there were no significant differences among other groups at 4 weeks (*P* > 0.05). The serum levels of OPG were highest in the A group at 8 and 12 week time points (*P* > 0.05). At 8 and 12 weeks, the serum levels of OPG were 1.665 ± 0.597 ng/ml and 1.672 ± 0.476 ng/ml in A group rats, and there were no significant difference with the other groups (*P* > 0.05).

## Discussion

Although the pathology of Kashin-Beck disease may be explained by the effect of mycotoxins, and different species of fungi produce a wide range of mycotoxins, low nutrition diet may be involved in the aetiology of KBD [10],[28]-[30].The A'ba region of Sichuan Province is an endemic area of KBD. It is located on the Tibetan plateau, which has a high altitude and high humidity and a low temperature region. The rat fed with KBD-affected diet shows focal necrosis and irregular premature closure at epiphysis plate by microscopy examination. The pathology of tibia growth cartilage and adjoining metaphyseal bone showed T-2 toxin combined with low nutrition diet which retarded the growth of rats similar to the changes of epiphysis plate with deep necrosis in KBD, and the necrotic lesions in epiphysis cartilage were multiple and localized in the deep zone of epiphyseal plate. The basic pathological change of KBD is the epiphyseal plate showing focal or irregular premature closure, which means that the normal bone formation of endochondral ossification was destroyed. However, the precise mechanism of chondrocyte necrosis and epiphyseal plate destruction in KBD remains unknown [31],[32].

KBD-affected diets and low nutrition diet, which was made on the basis of the KBD-affected family dietary pattern which lacks some of the nutritional elements, will lead to cell division, proliferation and self-repair capacity decline [28],[33]. Based on epidemiological investigations, mycotoxins are significantly associated with the risk of KBD [10]. Cereal samples in high incidence areas of KBD were found to be more seriously contaminated with trichothecenes when compared to those in low incidence areas [20]. Through the metaphyseal rich, open capillary network, T-2 toxin can inhibit the proliferation of cartilage cells and reduce synthesis of proteoglycan and collagen. Although T-2 toxin can induce the chondrocyte necrosis in animals, which bind to the eukaryotic 60 S ribosomal subunit causing inhibition of protein, DNA and RNA synthesis, it has no target cell specificity. Thus, the mycotoxin, which was seen as important environmental factors in the pathogenesis of Kashin-Beck disease, may have more complex network in the body, especially in the blood and bone marrow system [10]. The serum levels of M-CSF in KBD-affected diet group rats and low nutrition diet combined with T-2 toxin group rats were higher than those in the other groups, which means that the T-2 toxin not only induce the chondrocyte necrosis but also affect the metabolic process of M-CSF [34].

From the basic pathological change of epiphyseal plate showing focal or irregular premature closure in KBD, the destroyed bone formation of endochondral ossification that is investigated may be a key for a KBD study [30]. Recent studies have revealed an essential role of M-CSF and RANKL in the development of osteoclasts, and detailed molecular cascades that induce osteoclast differentiation, activation and apoptosis have been clarified [25]. Molecular mechanisms of RANK/RANKL/OPG are keys in osteoclast differentiation and activation in physiological and pathological conditions. When RANKL and M-CSF bind their receptors RANK and c-fms, respectively, osteoclast precursor cells differentiate into osteoclasts [27],[35],[36]. KBD-affected diets and low nutrition diet, which was made on the basis of the KBD-affected family dietary pattern that lacks some of nutritional elements, will lead to cell division, proliferation and self-repair capacity decline. The serum levels of RANKL were highest in the KBD-affected diet group rats.

The suppressed immunity and chronic hypoxia-induced mitochondrial damage and apoptosis may play an important role in the pathogenesis of KBD [37]-[39]. OPG interferes with the interactions between RANK and RANKL and inhibits osteoclast formation and function. It has been shown that the RANKL/OPG ratio may determine the delicate balance between bone resorption and synthesis [27]. Under normal conditions, RANKL is mainly produced by osteoblasts and bone marrow stromal cells. However, in many pathological conditions such as rheumatoid arthritis (RA) and neoplastic osteolysis, RANKL is also produced by T and B lymphocytes, macrophages/monocytes, fibroblasts, synoviocytes and megakaryocytes. T-2 toxin and its accumulating effect may cause a decrease in the normal rate of osteoprogenitor cell differentiation to osteoblasts and osteocytes [40]. The serum levels of OPG were highest in the KBD-affected group rats. Based on these previous studies [3]-[9], the aetiology of KBD not only causes epiphyseal plate chondrocyte necrosis but also decreases epiphyseal plate bone formation both by decreasing the rate of bone formation and increasing the bone resorption. Thus, the mycotoxin, which are seen as exogenous free-radical carriers, are important environmental factors in the pathogenesis of Kashin-Beck disease [41],[42], and selenium, vitamin C and vitamin E, which inhibit free-radical formation, are considered to be protective [12],[33].

## Conclusion

The molecule of M-CSF, RANKL and OPG may be involved in the regulation of epiphyseal plate injury and repair in KBD, and its participation in the pathogenesis of KBD should be studied in the future.

## Competing interests

The authors declare that they have no competing interests.

## Authors' contributions

DY participated in the design of the study and drafted the manuscript. YS and FP participated in the design of the study and coordination and helped to draft the manuscript. All authors read and approved the final manuscript.
